# *Amphimermis enzoni* n. sp. (Nematoda: Mermithidae) parasitizing damselflies and dragonflies in Argentina

**DOI:** 10.21307/jofnem-2020-051

**Published:** 2020-05-18

**Authors:** José Matias Rusconi, Cristian Di Battista, Darío Balcazar, Matías Rosales, María Fernanda Achinelly

**Affiliations:** 1 Centro de Estudios Parasitológicos y de Vectores, (CEPAVE)-CCT-La Plata-CONICET-UNLP, Boulevard 120 S/N e/61 y 64, (1900) La Plata, Buenos Aires, Argentina; 2 Instituto de Limnología “Dr. Raúl A. Ringuelet”, (ILPLA) La Plata-CONICET-UNLP, Boulevard 120 S/N e/61 y 64, (1900) La Plata, Buenos Aires, Argentina

**Keywords:** Argentina, Mermithida, Odonata, Parasitism, Stream

## Abstract

A mermithid nematode was found parasitizing nymphs of dragonflies and damselflies. The host specimens were collected from the stream Cajaravilla, Magdalena, Buenos Aires state, Argentina. In this work, we described *Amphimermis enzoni* n. sp., a nematode new to science. Nematodes were identified through morphological and molecular methods. The combination of the following characters separates *A. enzoni* n. sp. from other members of the genus *Amphimermis* Steiner: long and S-shaped vagina, twisted spicules for proximal 34% of their length, untwisted for 12%, again twisted for 30%, and untwisted for the last 24%; genital papillae arranged in three rows, medial row marginally longer than sub-medial rows; medial row bifurcated immediately anterior and posterior to cloaca, with 111 genital papillae (73 pre-anals and 38 post-anals). The sequences of 18S rDNA regions from *A. enzoni* formed a well-supported monophyletic clade with two GenBank sequences of *Amphimermis* spp. (EF617354 and EF617355) with 0.63 to 1.26% divergence and two Mermithidae spp. (LC512371 and LC512370) with 0.63 to 1.1% divergence, respectively. To our knowledge, this is the first example of mermithid infection in nymphs of dragonflies and damselflies for South America.

Mermithids are obligate parasites of invertebrates. Most of them have been found to be parasites of insects, even though they are also found in spiders, crustaceans, leeches, and molluscs (Poinar, 1975; Poinar and Stockwell, 1988; Poinar and Ćurčić, 1992). In their life cycle, infective juveniles (preparasitic second-stage juveniles [J2]) hatch from eggs and actively seek and penetrate the host. The third-stage juvenile (J3) develops in the host, at which time the postparasitic fourth-stage juvenile (J4) emerges, killing the host. The J4 develops into adults, which mate and lay eggs in the substrate. Juveniles hatched from these eggs will penetrate a new host and begin the cycle anew (Becnel and Johnson, 1998).

While conducting an extensive survey for parasites of mosquitoes, nymphs of dragonflies and damselflies from a stream were found containing mermithid nematodes.

There are few articles about mermithid infections on Odonata worldwide. Artykhovsky and Negrobov (1967) discovered an undescribed mermithid in naiads of dragonflies in Usman, Russia, and Willis (1970) found an *Amphimermis* specimen in naiads of damselflies in the USA. These two reports are the only known records of mermithid nematodes in Odonata.

In Argentina, the genus *Amphimermis* was cited for the first time by Miralles and Camino (1983). They described the species *A. bonaerensis* parasitizing the grasshopper *Laplatacris dispar* Rhen, 1939. Camino and Lange (1997) found two new species of the genus, also parasites of grasshoppers: *A. ronderosi* in *Metaleptea brevicornis* (L.) (Orthoptera: Acrididae: Acridinae) and *A. dichroplusi* in *Dichroplus elongatus* Giglio-Tos, 1894 (Orthoptera: Acrididae: Melanoplinae).

In the present study, a new species of the genus *Amphimermis*, *Amphimermis enzoni* n. sp. is described from Argentina. To our knowledge, this is the first example of a mermithid infection in nymphs of dragonflies and damselflies for South America.

## Materials and methods

### Insect sampling and nematode collection

Nymphs of dragonflies and damselflies were found in the stream Cajaravilla, Magdalena (34° 47´ 25˝ S; 58° 08´ 55˝ W), Buenos Aires Province, Argentina. The insects were collected near flooded reeds with a scoop (400 ml), placed in individual recipients with water from the environment and taken to the laboratory. Nematodes were obtained from the emergence of parasitized insects and maintained on 9 cm diameter Petri dishes with sterilized soil (in oven at 220°C during 1 hr) and 40 ml of water from the same stream.

### Nematode identification

For the morphological description, the mermithids were fixed in T.A.F. (2% triethanolamine, 7.5% formaldehyde in distilled water). All measurements are in micrometers unless otherwise specified and are presented as the range followed by the mean in parentheses. Specimens for molecular studies were fixed in absolute ethanol. Nematodes were measured using an ocular micrometer in a Leica DM 500 microscope. Photographs were taken with an Olympus DP-71 camera and a Leica DM 500 microscope; drawings were made using an Olympus CX-31 with drawing tube. For the examination of the hypodermal chords, a 0.3 to 0.5 cm portion of the midbody region of two specimens was removed and slide-mounted in a glycerin-lactic acid stain for observation at 10-100 x according to Tripodi and Strange (2018).

Voucher specimens have been deposited in the Museo de Ciencias Naturales de La Plata, Buenos Aires, Argentina.

For the scanning electron microscopy (SEM) study, specimens were dehydrated through a graded series of ethyl alcohols and critical point dried with carbon dioxide; then specimens were mounted on metal stubs with silver paste, coated with gold, and examined in a Philips 505 scanning electron microscope equipped with a digital imaging program (Philips Electron Optics BV, Eindhoven, Netherlands).

To confirm the nematodes identification, a molecular approach was performed. Genomic DNA was extracted using 100 μl of a 5% suspension of Chelex in deionized water and 2 μl of proteinase K, followed by overnight incubation at 56°C, boiling at 90°C for 8 min and centrifugation at 14,000 rpm for 10 min. An aliquot of 1 μl of the supernatant was utilized as template for Polymerase Chain Reaction (PCR). The 18S rRNA partial sequences were amplified using the primers Merm F 18S (5´-CAAGGACGAAAGTTAGAGGTTC-3´) and Merm R 18S (5´-GGAAACCTTGTTACGACTTTTA-3´) according to Kobylinski et al. (2012) with the Go Taq Master Mix (Promega Corporation, Madison, USA). The thermocycle conditions were as follows: 94°C for 15 min; 35 cycles of 94°C denaturation for 30 s, annealing 52°C for 40 s and extension 72°C for 60 s; a single final extension period of 72°C for 10 min. PCR products were analyzed by electrophoresis on 1% agarose gels and visualized by staining with ethidium bromide. The amplicons were sequenced in Macrogen Inc. (Korea) and edited with the platform GENEIOUS (Biomatters, Auckland, NZ, http://www.geneious.com) (Kearse et al., 2012). The consensus sequences obtained were compared with sequences in the BLAST tool available in the NCBI database (http://www.ncbi.nlm.nih.gov). The resulting sequences were submitted to the National Center for Biotechnology Information (NCBI) GenBank database (http://www.ncbi.nlm.nih.gov) under the accession number MT021436. The evolutionary distances were computed by Bayesian inference (BI) analysis using MRBAYES Software package (Huelsenbeck and Ronquist, 2001) and the maximum composite likelihood method (Tamura et al., 2004) with the IQ-Tree software (Trifinopoulos et al., 2016). For the maximum composite likelihood the evolutionary model was estimated with the jModelTest 2 and the best-fit model was determined according to BIC criterion: TIM2e + I + Γ (Darriba et al., 2012).

## Results

In total, 17 specimens of *Amphimermis enzoni* n. sp. (11 juveniles, 3 ♀♀ and 3 ♂♂) were found in the cavity of 161 examined nymphs of *Ischnura fluviatilis* Selys, 1876 (prevalence 9.94%) and in 21 of *Rhionaeschna bonaerensis* Rambur, 1842 (prevalence 4.76%). The mean intensity of infection was 1.14 (1-2) parasite specimens per infected host of *I. fluviatilis* and 1 (0-1) per infected host of *R. bonaerensis*.

**Figure 1:**
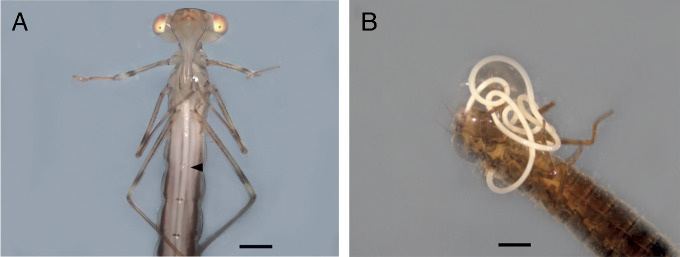
A. Nematode inside *I. fluviatilis*. (arrowhead) B. Post-parasitic juvenile (J4) emerging from *R. bonaerensis*. Scale bars: 1 mm.

As the nematodes matured, they oriented themselves longitudinally in the abdominal and thoracic regions of the host and could be seen with the naked eye during the late stages of parasitic development (Fig. 1A). The nematodes emerged from the hosts in the regions of the anus or mouth (Fig. 1B) and this escape did not always immediately kill the host. Some hosts (*n* = 5) were able to live between 2 and 7 days until dead.

### *Amphimermis enzoni* n. sp.

**Figure 2:**
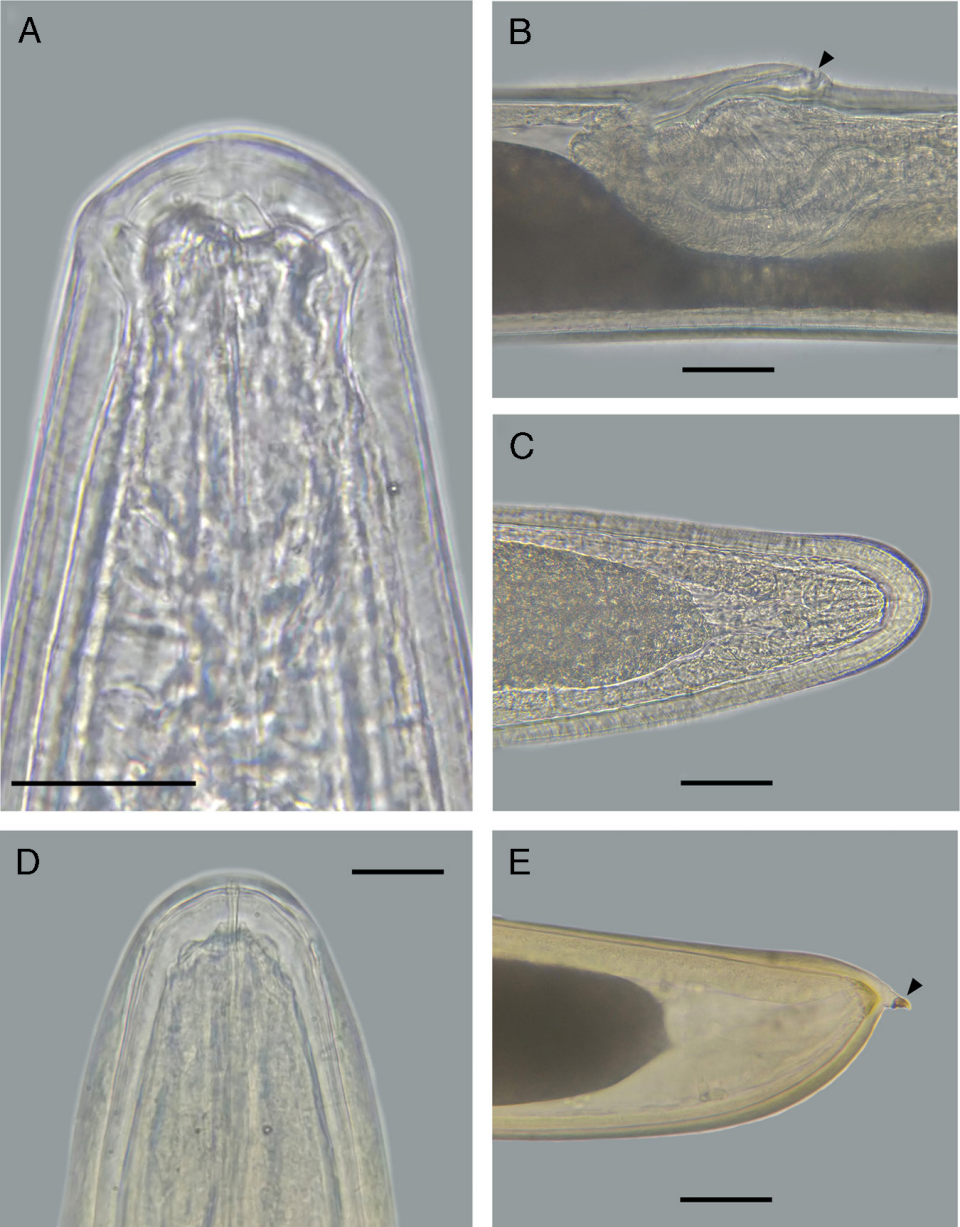
Female (A-C). A. Head. B. Vulva (arrowhead). C. Tail. Post-parasitic juvenile (D, E). D. Head. E. Tail with appendage (arrowhead). Scale bars: 50 μm.

**Figure 3:**
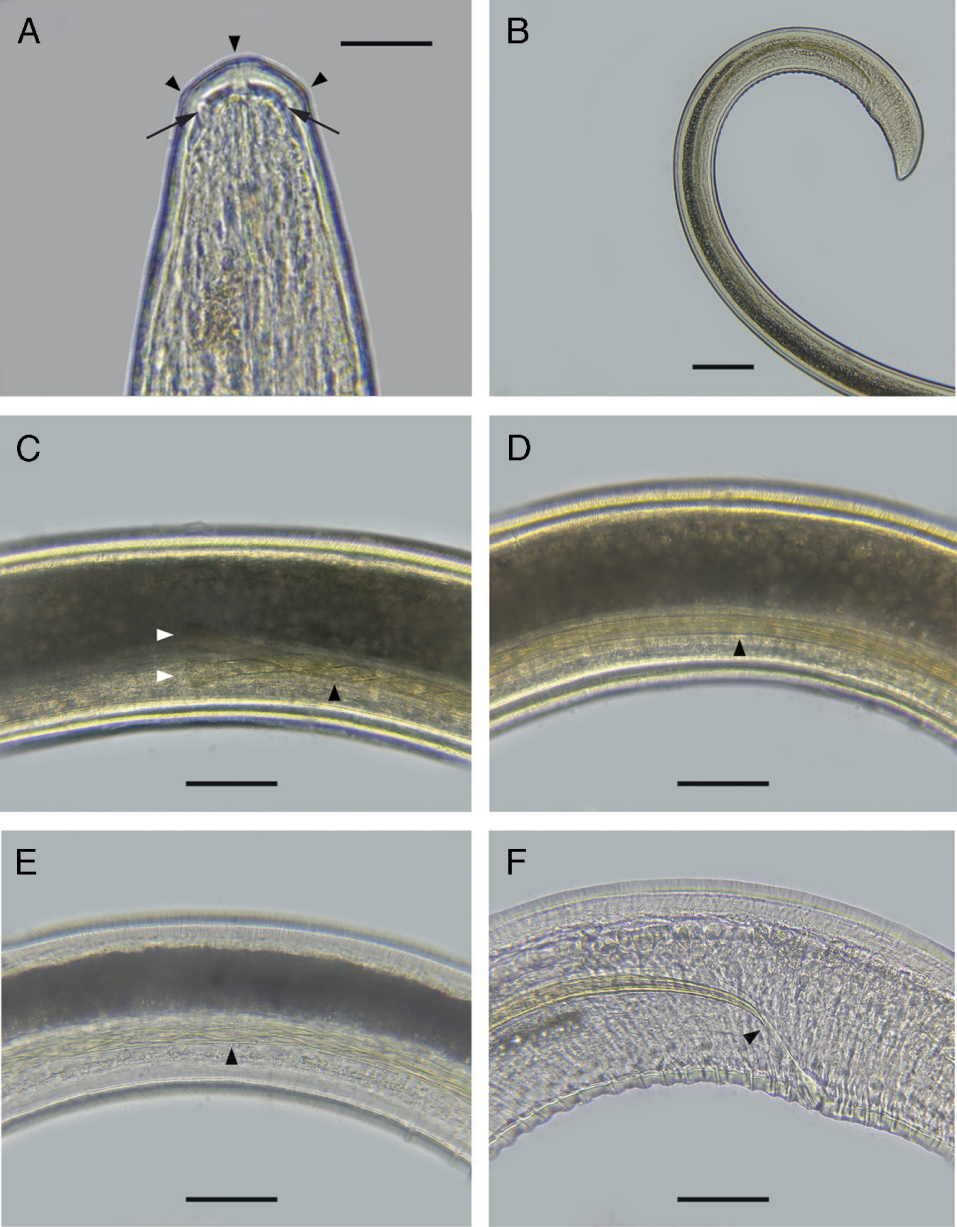
Male (A-F). A. Head showing cephalic papillae (arrowheads) and amphids (arrows). B. Tail showing the spicule. C. Proximal part of the spicule (white arrowheads showing the beginning of the spicule and black arrowhead showing a section of the proximal twisted part). D. Untwisted part of the spicule. E. Distal twisted part of the spicule (arrowhead). F. Tip of the spicule (arrowhead). Scale bars: 50 μm (A-F), 200 μm (B).

**Figure 4:**
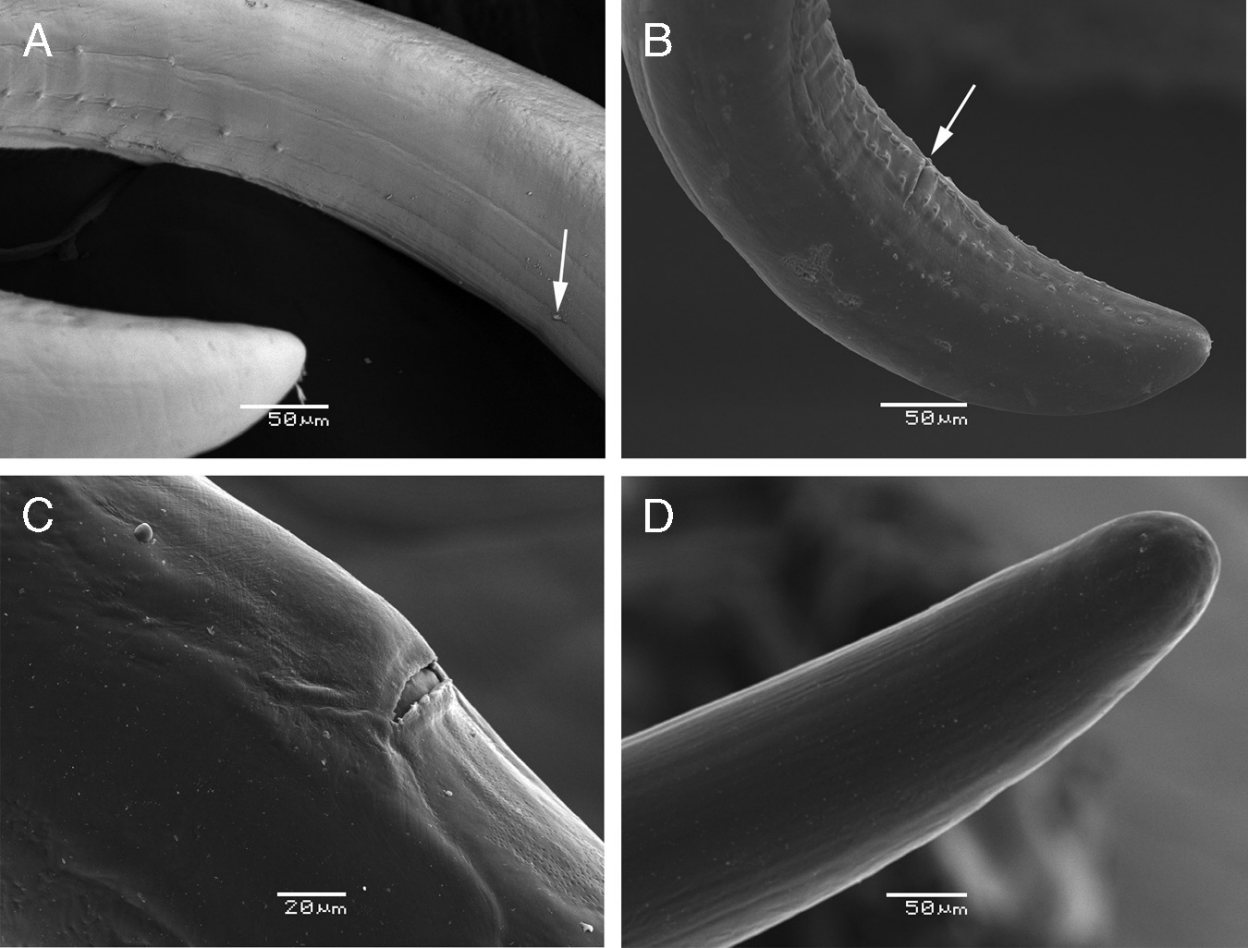
Male tail (A, B). A. First papilla separated from the rest (arrow) of the cephalic papillae. B. Cloaca (arrow). Female (C, D). C. Vulva. D. Tail.

**Figure 5:**
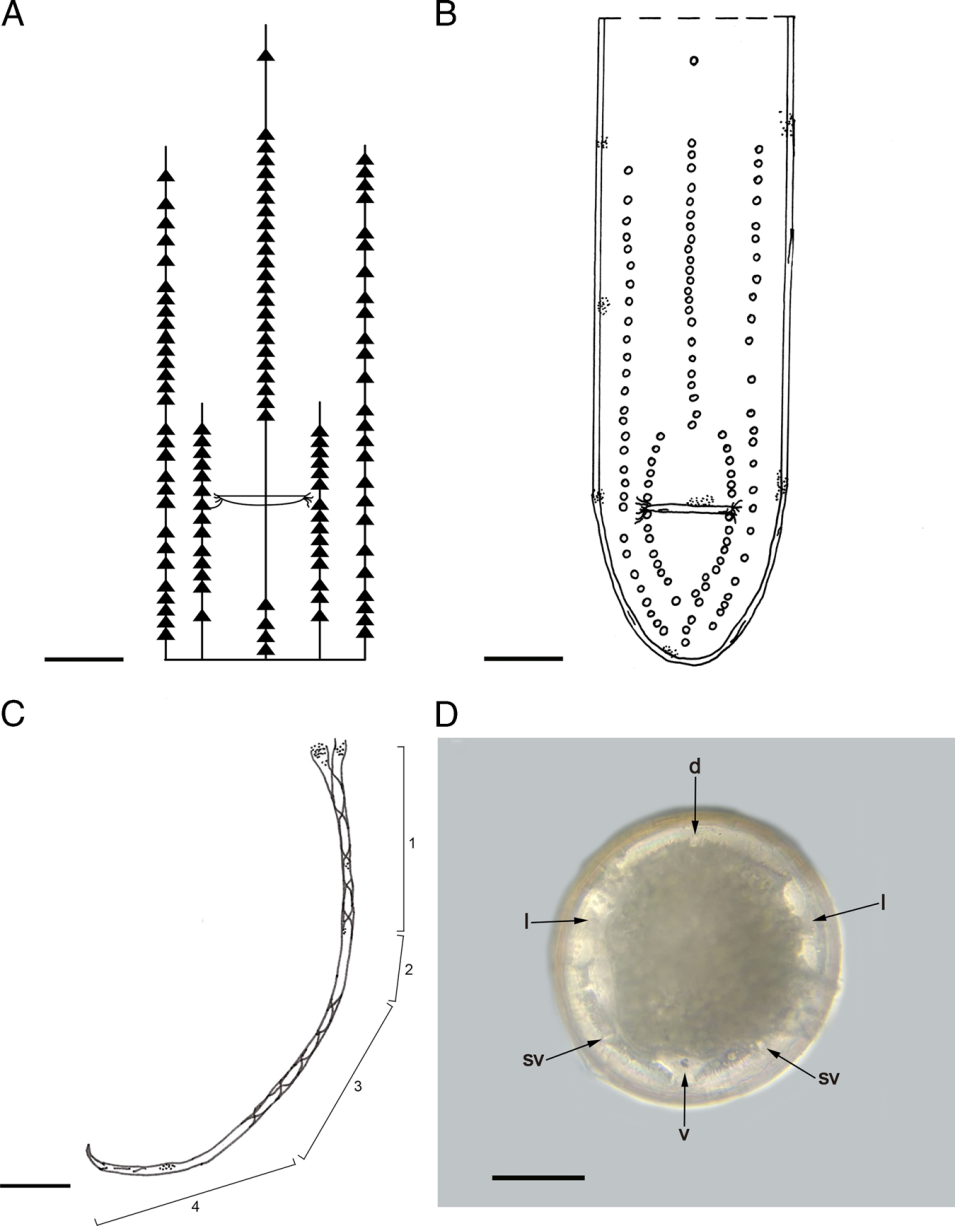
Male (A-D). A. Schematic arrangement of genital papillae. B. Tail, ventral view showing arrangement of genital papillae. C. Scheme of the spicule: 1 (proximal twisted part), 2 (untwisted part), 3 (distal twisted part), 4 (tip). D. Cross section, mid-body. d, dorsal chord; l, lateral chord; sv, subventral chord; v, ventral chord. Scale bars: 50 μm (A-C), 9 μm (D).

(Figs. 2A-E, 3A-F, 4A-D, 5A-D).

### Measurements

Female (*n* = 3) has length 72.6 ± 2.8 (47-103) mm, greatest width 263 ± 48.03 (216-312) μm, width of the head at the level of the cephalic papillae 57 ± 5.1 (54-63) μm, width of the body at the level of the nerve-ring 141 ± 18.7 (135-162) μm, distance anterior extremity to nerve-ring 312 ± 59.9 (243-351) μm, distance anterior extremity to amphidial pouch 36 μm, diameter of amphidial opening 18 μm, width at the level of the trophosome 198 ± 38.2 (171-225) μm, width of vagina 135 ± 12.2 (126-144) μm, length of vagina 795.5 ± 4.9 (792-799) μm, width of vulva 31.5 ± 6.4 (27-36) μm, length of vulva 103.5±6.4 (99-108) μm, and tail length 229.5 ± 70 (180-279) μm.

Male (*n* = 3) has length 38±0.36 (34-41) mm, greatest width: 210 ± 10.3 (198-216) μm, width of the head at the level of the cephalic papillae 72 ± 15.6 (54-81) μm, width of the body at the level of the nerve-ring 126 ± 9 (117-135) μm, distance anterior extremity to nerve-ring 252 ± 31.2 (216-270) μm, distance anterior extremity to amphidial pouch 27 μm, diameter of amphidial opening 18 μm, width at the level of the trophosome 132 ± 5.2 (126-135) μm, length of spicules 1286.3 ± 71 (1215-1357) μm, width of the body at middle of spicules 118 ± 10.4 (162-180) μm, body width at cloacal opening 162 ± 9 (153-171) μm, and tail length 225 ± 9 (216-234) μm.

Post-parasitic juvenile (*n* = 11) has length 65.5 ± 14.9 (47-84) mm, greatest width 250.36 ± 32.9 (207-306) μm, width of the head at the level of the cephalic papillae 76 ± 4.74 (72-81) μm, distance anterior extremity to nerve ring 234.87 ± 51.23 (162-315)  μm, width of the body at the level of the nerve-ring: 167.62 ± 34.66 (126-243) μm, tail length 300.6 ± 119.86 (216-522) μm, and tail width 198.81 ± 30.77 (144-252) μm.

### Description

#### General

They are white medium size nematodes vary from 3 to 10 cm. Cuticle has cross fibers. Head is rounded. Mouth is terminal. There are six cephalic papillae around the mouth. Opening of lateral cephalic papillae is at the level of or slightly anterior to the level of sub-median cephalic papillae. Amphids are cup-shaped. There are six hypodermal cords.

#### Female

In female, opening of the vulva is a transverse slit at the middle of the body. Vulval flap is present. Vulval cone is absent. Vagina is S shaped, long, muscular and posterior loop is slightly smaller in length than anterior loop and is bended rounded. The junction of vagina and uterus is slightly posterior to vulva. Tail is conical and slightly flattened on the ventral surface.

#### Post-parasitic juvenile

They are similar in size to the adults. Head is rounded. Cuticle has cross-fibers and tail has appendage.

#### Male

In male, tail is curled, conoid, and bluntly rounded. Spicules are paired; proximal part is twisted for 34% of its length, then untwisted for 12%, twisted for 30%, and finally untwisted for 24%. First genital papillae are located at the level of the first untwisted part of the spicule, separated from the rest. Spicule length is approximately 8 × body width at cloaca. Genital papillae are arranged in three rows; medial row marginally longer than sub-medial rows; medial row is bifurcate immediately anterior and posterior to cloaca. There are a total of 111 genital papillae (73 pre-anals and 38 post-anals).

### Taxonomic summary

Following is the taxonomic summary:

Type host: *Ischnura fluviatilis* Selys, 1876 and *Rhionaeschna bonaerensis* Rambur, 1842.

Type locality/collection dates: stream Cajaravilla, Magdalena (34° 47´ 25˝ S; 58° 08´ 55˝ W), Buenos Aires Province, Argentina; October to February 2018-2019; 2019-2020.

Site of infection: body cavity.

Prevalence: 9.94% in *Ischnura fluviatilis* and 4.76% in *Rhionaeschna bonaerensis.*

Specimens deposited: all types have been deposited in the Colección Helmintológica del Museo de Ciencias Naturales de La Plata with the following accession numbers: Holotype male No. MLP-He 7639, allotype female No. MLP-He 7640, and one paratype (postparasitic juvenile) No. MLP-He 7641.

Etymology: this species is named after Enzo Rusconi, nephew of JMR.

### DNA characterization and phylogenetic analysis

**Figure 6:**
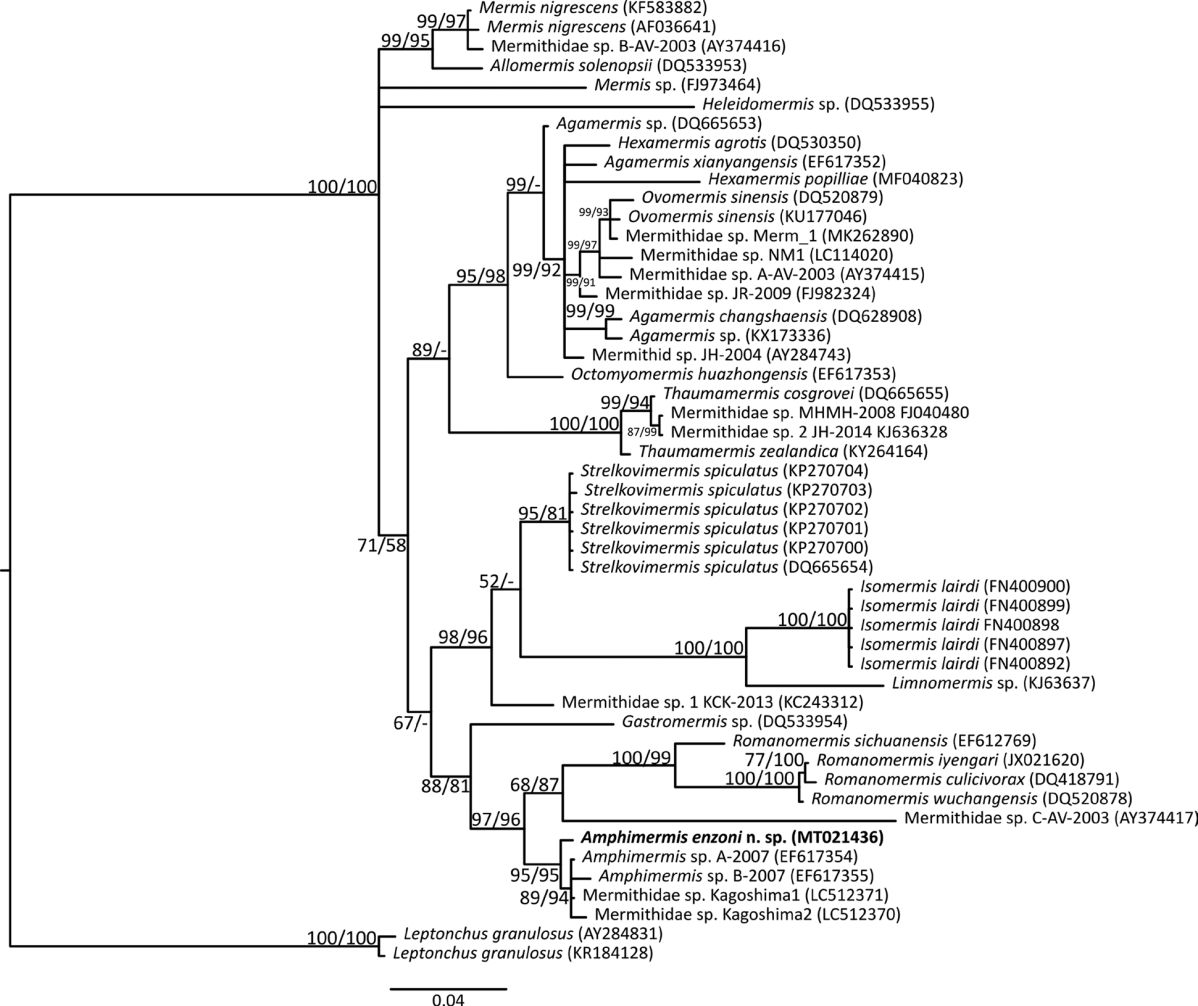
Phylogeny of the Mermithidae family based on 18S rDNA data including the new species, *Amphimermis enzoni.* The tree topology is inferred by Bayesian inference (BI) and maximum likelihood (ML). Posterior probability and ultrafast bootstrap values are reported as BI/ML. Only node consistencies above 50% are shown. The tree is drawn to scale, with branch lengths in the same proportions as the evolutionary distances used to infer the phylogenetic tree.

The 18S rDNA sequence of *Amphimermis enzoni* obtained (MT021436) was 633 bp. Specimens were analyzed by Blast matched sequences with two GenBank sequences of *Amphimermis* spp. (EF617354 and EF617355) with 0.63 to 1.26% divergence and two sequences of Mermithidae spp. (LC512371 and LC512370) with 0.63 to 1.1% divergence, respectively (Fig. 6). The same tree topology for this group was observed with both Bayesian inference and maximum composite likelihood method.

## Discussion

*Amphimermis enzoni* n. sp. is characterized by its S-shaped type vagina, the twisting length of the spicule, a total of 111 genital papillae (73 pre-anals and 38 post-anals) and the arrangement of genital papillae in three rows, medial row marginally longer than sub-medial rows and medial row bifurcate immediately anterior and posterior to cloaca. Baker and Poinar (1994) divided the genus into four groups of species on the basis of the twisting of the spicules and the shape of the amphids. *Amphimermis enzoni* n. sp. is within the *bogongoe* group (spicules twisted loosely distal and proximal half, untwisted in the middle; cup shaped amphids) along with *A. bonaerensis*, *A. bongongoe* Welch, 1963, *A. dichroplusi*, *A. maritima* Rubstov, 1971, *A. mirabinda* (Baker and Poinar, 1994), *A. litoralis* Artykhovski and Karchenko, 1971, *A. ronderosi, A. thezamica* (Gorgadze et al., 2017), and *A. tinyi* Nickle, 1972 (Miralles and Camino, 1983; Camino and Lange, 1997; Gorgadze et al., 2017). *Amphimermis tinyi* was the only species of the genus found in Odonata (*Ischnura posita* Hagen and *Anomalogrion hastatum* Say) until this contribution.

The male of *A. enzoni* n. sp. differs from all the species in the group by the twisted length of the spicules and the number and arrangement of the genital papillae. Spicule length of *A. enzoni* n. sp. is comparable to that of *A. mirabinda* and *A. ronderosi.* However, *A. mirabinda* can be distinguished from the species described here in having enormous amphids*. A. ronderosi* is differentiated from *A. enzoni* n. sp. in the twisting and untwisting percentage of the spicule length (41, 14, 31, and 14% vs 34, 12, 30, and 24%).

Female of *A. enzoni* n. sp. is distinguished from *A. bogongoe*, *A. bonaerensis*, *A. thezamica*, and *A. ronderosi* females in having a longer vagina (792-799 vs 160-205, 51-113, 180-270 and 100-350 μm, respectively); it has shorter vagina length compared to that of *A. mirabinda* and *A. dichroplusi* which can reach up to 975 and 2985 μm, respectively.

Genetically, 18S sequences from these specimens matched known representatives of *Amphimermis* with <1.2% divergence. Hitherto, there have not been molecular analyses in the descriptions of species of the genus *Amphimermis.*

*Amphimermis enzoni* n. sp. clustered with the only known *Amphimermis* sp. sequence and also to two unidentified mermithids isolated from the shield bug *Parastrachia japonensis* (Hemiptera) (LC512371 and LC512370) as well as two *Amphimermis* sp. (EF617354 and EF617355). None of the *Amphimermis* sp. sequences within this clade have originated from organisms reported identities.

This paper contributes the first molecular characterization of a species of the genus *Amphimermis.* To our knowledge, this is the first example of mermithid infection in nymphs of dragonflies and damselflies for South America.
